# Fear of hypoglycemia and sleep in children with type 1 diabetes and their parents

**DOI:** 10.3389/fendo.2024.1419502

**Published:** 2024-12-16

**Authors:** Ulrike Schierloh, Gloria A. Aguayo, Muriel Fichelle, Cindy De Melo Dias, Anna Schritz, Michel Vaillant, Katharine Barnard-Kelly, Ohad Cohen, Inge Gies, Carine de Beaufort

**Affiliations:** ^1^ Department of Pediatric Diabetes and Endocrinology, Clinique Pédiatrique, Centre Hospitalier, Luxembourg, Luxembourg; ^2^ Deep Digital Phenotyping Research Unit, Department of Precision Health, Luxembourg Institute of Health, Strassen, Luxembourg; ^3^ Competence Center for Methodology and Statistics, Luxembourg Institute of Health, Strassen, Luxembourg; ^4^ Southern Health NHS Foundation Trust, Southampton, United Kingdom; ^5^ Institute of Endocrinology, Sheba Medical Center, Tel Hashomer, Israel; ^6^ Division of Pediatric Endocrinology, Department of Pediatrics, University Hospital Brussels, Brussels, Belgium; ^7^ Research Unit Research Group Growth and Development (GRON), Vrije Universiteit Brussel, Brussels, Belgium

**Keywords:** type 1 diabetes (T1D), children, parents, fear of hypoglycemia, sleep, sensor augmented pump, iscCGM

## Abstract

**Aims:**

To compare impact of pump treatment and continuous glucose monitoring (CGM) with predictive low glucose suspend (SmartGuard) or user initiated CGM (iscCGM) on sleep and hypoglycemia fear in children with type 1 Diabetes and parents.

**Methods:**

Secondary analysis of data from 5 weeks pump treatment with iscCGM (A) or SmartGuard (B) open label, single center, randomized cross-over study was performed. At baseline and end of treatment arms, sleep and fear of hypoglycemia were evaluated using ActiGraph and questionnaires.

**Results:**

31 children (6-14 years, male: 50%) and 30 parents (28-55 years) participated. Total sleep minutes did not differ significantly for children (B vs. A: -9.27; 95% CI [-24.88; 6.34]; p 0.26) or parents (B vs. A: 5.49; 95% CI [-8.79; 19.77]; p 0.46). Neither daytime sleepiness nor hypoglycemia fear in children or parents differed significantly between the systems. Neither group met recommended sleep criteria.

**Conclusion:**

Lack of sleep and fear of hypoglycemia remain a major burden for children with diabetes and their parents. Whilst no significant differences between the systems were found, future technology should consider psychosocial impacts of diabetes and related technologies on children and parents’ lived experience to ensure parity of esteem between physical and mental health outcomes.

**Clinical Trial Registration:**

www.ClinicalTrials.gov, identifier NCT03103867.

## 1 Introduction

The daily management of type 1 diabetes (T1D) is a 24/7 challenge for children and their caregivers and may have a major negative impact on their sleep and quality of life (1–3).

Fear of nocturnal hypoglycemia is common and a significant concern amongst parents of children with T1D (4–6), and is associated with enhanced attention to frequent checking of their children’s glycemia or sensor values or to get up during the night (7, 8). Data show that fear of hypoglycemia can lead to chronic sleep disturbance for the parents as for their children with diabetes (9–11). This highly prevalent chronic sleep interruption can affect caregivers of children with T1D with negative effects on their daily functioning and well-being (12, 13).

New technologies have been introduced to facilitate and improve care with automated sensor-augmented pump (SAP) and predictive low glucose suspend and alerts (SmartGuard) or with user-initiated intermittently scanned continuous glucose monitoring (iscCGM, Freestyle libre).

SAP treatment leads to improved metabolic outcome (14). Alerts about hypo-and hyperglycemia are programmed in SAP in order to enable patients and their caregivers to react quickly to such information. The Minimed 640G pump with SmartGuard feature combines alerts with an automated insulin suspension to prevent hypoglycemia. The pump suspends insulin infusion when the sensor glucose (SG) is within 3.9 mmol/l (70 mg/dl) above the low limit and predicted to be 1.1 mmol/l (20 mg/dl) or lower above the low limit in 30 min. Glucose values and glucose trends are available on the pump screen (15).

A multicenter evaluation shows that SmartGuard technology significantly reduces the risk for hypoglycemia in pediatric diabetes patients without increasing HbA1c (16).

However, alerts may be perceived as intrusive and anxiety-inducing which can lead to diabetes distress and alert fatigue as well as nocturnal awakenings (8, 17).

Freestyle Libre 1 is a device measuring the interstitial glucose levels continuously. The results can be obtained when the patient/caregiver actively scans the sensor (iscCGM): no alerts are given for hypo-or hyperglycemic events, nor is information available when the sensor is not scanned. Data is lost when more than 8 hours elapse between scans. No communication exists between this glucose measurement and the insulin pump (15).

The evaluation of iscCGM being as safe as self-monitoring of blood glucose (SMBG) and having a better metabolic outcome than SMBG is demonstrated in children (18, 19).

The impact of these technologies on metabolic control has been studied before (20).

To our knowledge, no study has yet addressed the focus on comparing the impact of these two technologies on fear of hypoglycemia, quality and quantity of sleep in children and their caregivers. In this report we analyze these questions using questionnaires, sleep diaries and ActiGraph data in the QUEST trial (15).

## 2 Materials and methods

### 2.1 Ethics committee statement

This study was approved by the National Luxembourgish Ethics committee (CNER). Only pseudonymized data was used for the analysis.

### 2.2 Study design and randomization

The study had an open-label, single-center, randomized, two-period crossover design.

Each patient was randomly allocated; the sequence codes (A-B or B-A) were determined in advance (15).

### 2.3 Participants

Patients fulfilling the following inclusion criteria got included: age between 6 and 14 years, type 1 diabetes and on insulin pump treatment for at least 6 months and HbA1c ≤ 11% (≤ 96.72 mmol/mol) (30).

Exclusion criteria were physical or psychological disease likely to interfere with an appropriate conduct of the study and chronic sleep medication used by the patient or by the participant primary caregiver. Prior to enrollment, written informed consent was obtained from the parents and all children gave their informed assent (30).

### 2.4 Procedures

The participants were randomized either to treatment A, insulin pump Minimed 640G and independent iscCGM (Freestyle libre 1) or to treatment B, SAP with SmartGuard feature (Minimed 640G), each for 5 weeks. Following a 3 weeks washout period the participants crossed over to the other study arm for another 5 weeks.

No specific dietary advice was given.

The week before randomization as well as during the last 7 days of each treatment the participants and one of their caregivers (same reference person throughout the course of the study) wore a sleep device on the wrist (ActiGraph) and completed a sleep diary. Before the start and at the end of each treatment arm the subject and his caregiver were asked to fill in the questionnaires.

To evaluate the hypoglycemia fear, the Children’s Hypoglycemia Survey (CHS) and Hypoglycemia Fear survey for parents were used. The Children’s Hypoglycemia Survey (24 items) measures 3 areas of hypoglycemia fear: their general fear of hypoglycemia and its consequences, the children’s fear of hypoglycemia in a specific situation, and the children’s behavior to avoid hypoglycemia. The survey for parents is divided into 2 subscales-scores, one asking about parental worry about their child’s hypoglycemia (15 items), and the other about behavior to prevent hypoglycemia for their child (11 items) (21–25).

Daytime sleepiness in the children and their caregiver were evaluated using the Epworth sleepiness scale, a self-administered questionnaire which provides a measurement of the subject’s general level of daytime sleepiness (26). The Epworth Sleepiness Scale is defined based on questions about the chances to fall asleep in different situations. The score ranges from 0-24 with the following interpretation: score 0-5: lower normal daytime sleepiness/6-10: higher normal daytime sleepiness/11-12: mild excessive daytime sleepiness/13-15: moderate excessive daytime sleepiness/16-24: severe excessive daytime sleepiness (26).

The detailed conduct of the study was previously published (15).

The use of the two glucose measurement tools and the features of the Minimed 640G pump were explained during the dedicated training visit. All participants had access to a 24/7 diabetes hotline in case of technical or any other issues. Settings of the SmartGuard were standardized based on the current experience (20). The low limit was set at 3.4 mmol/l, with an insulin suspension at ≤7.3 mmol/l if the predicted value within 30 minutes was 4.5 mmol/l (15).

### 2.5 Methods

Randomization (ratio 1:1) was performed by a statistician with 4 blocks of 8 participants and equal treatment allocation based on prepared envelopes with the sequence code (A-B or B-A). In this randomized block design the sequence codes were randomly allocated to each block. This kind of design is used to minimize the effects of systematic error.

After consenting, the envelope was opened by the medical team to provide the participant with the allocated treatment sequence (15, 30).

Blinding was not possible for the participant nor the medical team.

A sample size of 36 patients with a minimum of 31 patients was calculated for a power of 80% (15).

To ensure data quality, double data entry was performed within Ennov Clinical software, and online logical controls were performed with correction of erroneous data values.

Hypoglycemia Index in children (subscales and Hypoglycemia Fear Survey in parent/caregiver (subscales for hypoglycemia worry and behavior) at baseline and at the end of each treatment arm were also analyzed by using a linear mixed model with the same independent parameters as described previously.

Total sleep (minutes) and total wake time (minutes) and number of awakenings during the last 7 days of each treatment arm were measured by ActiGraph, in children and in one of their caregivers. Sleep analysis was performed using ActiLife data analysis software. The detailed assessment of sleep patterns was previously published (15). The average sleep time per night for each visit was used as the outcome to compare the two different treatments. Additionally, the average number of awakenings and the average length of total wake time per night and visit was compared between the two devices. Where Actigraph measurement of sleep was divided into more than one sleep period (due to being awake and getting out of bed for more than 10 minutes), total sleep time (defined by ActiLife), time and number of awakenings (defined by ActiLife), number of get-ups (number of sleep periods - 1), and time of being out of bed (time from out of bed till sleep onset) was added up to have one measurement per night. Sleep time during the day (nap; went to bed between 12pm and 7pm) was excluded from the analysis. For number of awakenings and total wake time, the estimations of the ActiLife algorithm (Sadeh for children (10-25 years) and Cole-Kripke for adults) were used as outcomes (27, 28).

Time to bed, time out of bed and number of awakenings were also compared with the sleep diary and some parameters were adjusted according to the sleep diary if they seemed too unrealistic when calculated by ActiLife. We used the ActiLife settings for bedtime (5 consecutive asleep minutes) and wake time (first 10 consecutive minutes of awake time following a sleep period). The definition of sleep is based on the accelerometer data. If there is no movement for at least 5 minutes, the period is defined as sleep. Vacation time was not taken into account.

Characteristics of children and parents were presented using mean and standard deviations (SD) for continuous variables, median, 25% and 75% quartiles (Q1, Q3) for count variables and frequency and percentage for categorical variables. Characteristics for children are shown for the total study population and separated by treatment sequences. Z score BMI are calculated with the formula Z score = (X-m)/SD; X = BMI, m = mean, SD = standard deviation of BMI of the reference population (WHO growth reference (2006) data) with same sex and age (29).

Total sleep, quality of sleep (Epworth sleepiness scale and sleep diary) and number of awakenings were analyzed by using a linear mixed model and a naïve Fisher’s Exact test with treatment given (A vs. B), treatment sequence (A-B vs. B-A) and period of treatment (week 5 vs. week 13) as fixed effects factors and patient as a random effect.

Least square means and their 95% confidence intervals (CI) from the linear mixed models were reported as adjusted mean average sleep time and adjusted average number of awakenings in children and parents.

All test were two-tailed and a p-value<0.05 was claimed statistically significant. RStudio 2021.09.2 was used for statistical analysis.

## 3 Results

32 children, 16 male (50%), between 6 -14 years with a mean HbA1c of 7.47%, (58.14 mmol/mol), SD 0.59, a mean diabetes duration of 5.91 years (SD 3.29), being on insulin pumps for 5.07 (SD 3.87) years, were included in this study. Metabolic outcome as primary endpoint was reported previously (30). One child dropped out of the study after the first visit at baseline, without wearing neither the ActiGraph nor filling out any of the questionnaires and sleep diaries. 31 children (16 males) completed the study.

30 caregivers (24 females, 28 – 55 years (mean 42.77 years, SD 5.96)) participated in the study.

One parent had two children included in the study, therefore only one questionnaire and sleep diary was filled out and one Actigraph was worn by the parent. 28 parents and children answered all the questionnaires.

**Table 1** (30) shows the demographic baseline values for study participants (31 children), **Table 2** the data for the participating parents (30 caregivers).

**Table 1:** Descriptive baseline characteristics of the participating children (30).

	Mean (SD)/N (%)		
	Total (N = 31)	Sequence A-B (N = 14)	Sequence B-A (N = 17)
**Age, years**	10.5 (2.3)	11.2 (2.2)	10.7 (2.5)
Gender			
**Female**	15 (48%)	7 (50%)	8 (47%)
**Male**	16 (52%)	7 (50%)	9 (53%)
Ethnicity			
**Caucasian**	30 (97%)	13 (93%)	17 (100%)
**African**	1 (3%)	1 (7%)	0 (0%)
**Height, cm**	143.7 (14.6)	145.6 (15.2)	142.1 (14.4)
**Weight, kg**	42.8 (13.2)	44.1 (15.7)	41.8 (11.2)
**BMI, kg/m^2^**	20.2 (3.1)	20.1 (3.9)	20.3 (2.5)
Z score BMI^a^	1.23 (0.6, 1.6)	1.2 (0.7, 1.5)	1.3 (0.6, 1.7)
**HbA1c, %**	7.5 (0.6)	7.6 (0.6)	7.3 (0.5)
**HbA1c, mmol/mol**	58.1 (6.5)	59.9 (6.9)	56.6 (5.9)
Diabetes duration, years^a^	5.6 (3.0, 8.2)	5.7 (3.7, 7.1)	5.6(2.9, 9.8)
Pump use, years^a^	4.0 (2.2, 8.3)	3.9 (2.4, 6.9)	4.5 (1.8, 9.1)

a Median (Q1, Q3).

Bold values indicate baseline characteristics.

**Table 2:** Descriptive baseline characteristics of the participating parents.

	Mean (SD)/N (%)
**N**	30
**Age, years**	42.8 (6.0)
Gender	
**Female**	24 (80%)
**Male**	6 (20%)
Ethnicity	
**Caucasian**	29(97%)
**African**	1 (3%)
**Height, cm**	168.0 (9.7)
**Weight, kg**	77.4 (17.0)
**BMI, kg/m^2^**	27.5 (6.2)

### 3.1 Description of missing data

#### 3.1.1 Children

For one child the glucose sensor values of the last visit are missing. For another child the questionnaire, sleep diary and ActiGraph data of the wash-out period are missing for visit 3. Two children did not return/fill out the questionnaire of visit 2. Two children did not completely fill out all questions at visit 3. The maximum percentage of missing data in the models was 6.5% for children (model including Epworth Sleepiness Scale).

#### 3.1.2 Parents

The parent of the child whose data were missing for visit 3, had also no data for visit 3. One parent had missing data for the ActiGraph and the sleep diary for visit 2, 3 and 4. Two parents did not fill out/return the questionnaire at visit 2. One parent did not answer one question at visit 3. The maximum percentage of missing data in the models was 10% for parents (model including Epworth Sleepiness Scale).

### 3.2 Sleep data results

#### 3.2.1 Total sleep time

Adjusted average sleep time for children in treatment A (pump and iscCGM) was 449.3 (95% CI [432.8; 465.7]) minutes per night (7.5 hours per night) and 440.0 (95% CI [423.6; 456.5]) minutes per night (7.3 hours per night) for treatment B (pump plus SmartGuard). No significant difference of total sleep time between devices was found (p-value 0.255).

For parents the adjusted average sleep time in treatment A was 413.8 (95% CI [395.4; 432.2]) minutes per night (6.9 hours) with a non-significant increase in sleep time of 5.5 minutes (95%CI [-8.8; 19.8]); p-value 0.46) for treatment B (419.3 (95% CI [400.9; 437.7])).

#### 3.2.2 Number of awakenings

The adjusted average number of nocturnal awakenings in children in treatment A (pump and iscCGM) was 24.7 (95% CI [22.5; 26.9]) and 25.2 (95% CI [23.0; 27.3]) in treatment B (pump plus SmartGuard); no significant difference between the devices was found (p-value 0.64).

For parents the adjusted average number of nocturnal awakenings in treatment A was 16.3 (95% CI [14.5; 18.1]) compared to 16.1 (95% CI [14.3; 17.9]) in treatment B; no significant difference between the devices and number of awakenings was found (p-value 0.76).

#### 3.2.3 Number of nocturnal get-ups

The adjusted average number of nocturnal get-ups in children did not show a significant difference between the two devices: 0.35 (95% CI [0.23; 0.48]) in treatment A compared to 0.41 (95% CI [0.28; 0.54]) in treatment B; p-value: 0.25. The number of nocturnal get-ups in parents was 0.58 (95% CI [0.36; 0.80]) in device A compared to 0.64 (95% CI [0.43; 0.86]) in device B; no significant difference was found (p- value 0.35).

### 3.3 Questionnaire results

#### 3.3.1 Hypoglycemia fear questionnaire

The score (Hypoglycemia Survey for children and for parent/caregiver with subscales for hypoglycemia worry and behavior) ranges from 0 = no fear to 104 high fear.

In the participating children, the adjusted mean for the hypoglycemia score was 55.1 (95% CI [51.7; 58.8]) for children in arm A. In treatment B, the score of hypoglycemia fear decreased by -0.8 (95% CI [-4.5; 2.9]), but no significant difference was observed between both devices (p-value = 0.67). In parents, the adjusted mean score was 40.67 (95% CI [33.1; 48.3]) in treatment A and decreased by -2.9 (95% CI [-7.0; 1.3]) points for device B. No significant difference for hypoglycemia fear was found between the devices (p= 0.18).

#### 3.3.2 Epworth sleepiness scale

The participating children showed on average a less normal daytime sleepiness during baseline, device A, B and washout period (summary in **Table 3**) than their parents (**Table 4**). No significant difference of the Epworth’s Sleepiness Scale and between device groups was found (p-value = 0.54). Also when only considering the 5-level interpretation scale of Epworth’s Sleepiness Scale with a naive Fisher’s Exact test, no significant differences was found between the devices (p = 0.90).

**Table 3:** Epworth’s sleepiness scale interpretation by device (N (%)), children’s answers.

	Baseline (N = 30)	Device A (N = 30)	wash-out (N = 29)	Device B (N = 28)
Lower normal daytime sleepiness	20 (67%)	17 (57%)	20 (69%)	17 (61%)
Higher normal daytime sleepiness	7 (23%)	7 (23%)	7 (24%)	7 (25%)
Mild excessive daytime sleepiness	1 (3%)	2 (7%)	0 (0%)	2 (7%)
Moderate excessive daytime sleepiness	2 (7%)	4 (13%)	1 (4%)	2 (7%)
Severe excessive daytime sleepiness	0 (0%)	0 (0%)	1 (4%)	0 (0%)

n, number of participants

**Table 4:** Epworth’s sleepiness scale interpretation by device (N (%)), parent’s answers.

	Baseline (N = 29)	Device A (N = 27)	wash-out (N = 29)	Device B (N = 27)
Lower normal daytime sleepiness	12 (41%)	10 (37%)	10 (35%)	12 (44%)
Higher normal daytime sleepiness	7 (24%)	9 (33%)	10 (35%)	9 (33%)
Mild excessive daytime sleepiness	4 (14%)	1 (4%)	4 (14%)	0 (0%)
Moderate excessive daytime sleepiness	2 (7%)	4 (15%)	1 (4%)	2 (7%)
Severe excessive daytime sleepiness	4 (14%)	3 (11%)	4 (14%)	4 (15%)

n, number of participants whose scores summed up to the respective sleepiness

## 4 Discussion

In our real-life study neither children with type 1 diabetes nor their parents show a significant difference in either hypoglycemia fear, quality or quantity of sleep during the use of two different glucose monitoring systems with or without the alarm function and predictive low glucose suspend. The lack of change in hypoglycemia fear may explain why we do not observe a change in sleep quality and quantity. Whether this depends on the short duration of our intervention or on other factors that were no taken into account is uncertain. The time to get used to a new system and develop a confidence in its function may vary between individuals and for some the 5 weeks may have been insufficient (31).

In our study, the mean sleep data outcome of our participants (children or caregivers) was below the recommended sleep duration as published by the American Academy of Sleep medicine (AASM). According to the Consensus Statement of the AASM children (6 to 12 years of age) should sleep 9 to 12 hours per 24 hours and teenagers (13 to 18 years) 8 to 10 hours per 24 hours to promote optimal health (32). Sleep deprivation occurs. when an individual fails to get enough sleep. In healthy children, sleep deprivation is associated with worse cognitive functioning, school performance and more behavioral problems (33).

In our study, the children, slept on average between 1.2 and 1.5 hours less than the minimum recommended sleep, in both treatment arms.

Per night, they slept an average of 9 minutes longer in treatment A (pump and iscCGM) compared to treatment B, which was not significant.

For adults, the AASM and the Sleep Research Society recommend in their Consensus Statement at least 7 or more hours per night on a regular basis to promote optimal health (34). The parents in our study missed on average the minimum of recommended sleep slightly (6.89 hours in treatment A and 6.98 in treatment B). Unlike their children, the parents in our study slept an average of 5.5 minutes longer per night in treatment B (pump and SmartGuard).

According to the consensus statements, all participants in our study are considered to be sleep deprived (children more than their parents).

Caregivers of children with T1D are known to be frequently sleep deprived and to worry about their child’s nighttime glucose (35). Sleep deprivation plays a role in different physiological processes influencing disease development (36). Treatment modalities, which can improve sleep quality and quantity, may have more impact on the general health and not only on diabetes outcome.

Sleep analysis and psycho-behavioral outcomes will have an added value in the evaluation of new technologies or new treatments and should be included as outcome parameter (37).

Future studies are needed to further explore the best use of new technologies and to offer a personalized medical approach.

## 5 Strength and limitations

The study is limited due to the constrained study duration and the number of participants. The study was powered for the primary outcome (percent of time spent in glucose target, TIT, (3.9 - 8 mmol/l) of treatment A and B during the final 7 days of the five-week device arm) (28).

The strength of the study derives from the fact that all data reflect the real world situation, as they were collected in free living at home. Another strength is the evaluation of sleep information with an objective method (Actigraphy) complemented with a sleep diary and not only based on self-reported data.

## Data Availability

The raw data supporting the conclusions of this article will be made available by the authors, without undue reservation.
